# Insights into the quantification and reporting of model-related uncertainty across different disciplines

**DOI:** 10.1016/j.isci.2022.105512

**Published:** 2022-11-05

**Authors:** Emily G. Simmonds, Kwaku Peprah Adjei, Christoffer Wold Andersen, Janne Cathrin Hetle Aspheim, Claudia Battistin, Nicola Bulso, Hannah M. Christensen, Benjamin Cretois, Ryan Cubero, Iván A. Davidovich, Lisa Dickel, Benjamin Dunn, Etienne Dunn-Sigouin, Karin Dyrstad, Sigurd Einum, Donata Giglio, Haakon Gjerløw, Amélie Godefroidt, Ricardo González-Gil, Soledad Gonzalo Cogno, Fabian Große, Paul Halloran, Mari F. Jensen, John James Kennedy, Peter Egge Langsæther, Jack H. Laverick, Debora Lederberger, Camille Li, Elizabeth G. Mandeville, Caitlin Mandeville, Espen Moe, Tobias Navarro Schröder, David Nunan, Jorge Sicacha-Parada, Melanie Rae Simpson, Emma Sofie Skarstein, Clemens Spensberger, Richard Stevens, Aneesh C. Subramanian, Lea Svendsen, Ole Magnus Theisen, Connor Watret, Robert B. O’Hara

**Affiliations:** 1 Department of Mathematical Sciences, Norwegian University of Science and Technology, Trondheim, Trøndelag 7034, Norway; 2 The Centre for Biodiversity Dynamics, Norwegian University of Science and Technology, Trondheim, Trøndelag 7491, Norway; 3 Department of Sociology and Political Science, Norwegian University of Science and Technology, Trondheim, Trøndelag 7491, Norway; 4 Kavli Institute for Systems Neuroscience and Centre for Neural Computation, Norwegian University of Science and Technology, Trondheim 7491, Norway; 5 Department of Physics, University of Oxford, Oxford, Oxfordshire OX1 3PU, UK; 6 Norwegian Institute for Nature Research, PO Box 5685, Torgarden, Trondheim, Trøndelag 7485, Norway; 7 Institute for Biology, Norwegian University of Science and Technology, Trondheim, Trøndelag 7491, Norway; 8 Bjerknes Centre for Climate Research, Bergen, Vestland 5007, Norway; 9 NORCE Norwegian Research Centre AS, Bergen, Vestland 5838, Norway; 10 The Department of Atmospheric and Ocean Sciences, University of Colorado Boulder, Boulder, CO 80309-0311, USA; 11 Peace Research Institute Oslo (PRIO), Oslo, Østlandet 0186, Norway; 12 KU Leuven, 3000 Leuven, Belgium; 13 Observatorio Marino de Asturias (OMA), Departamento de Biología de Organismos y Sistemas, University of Oviedo, 33071 Oviedo, Spain; 14 Department of Mathematics and Statistics, University of Strathclyde, Glasgow, Lanarkshire G1 1XH, UK; 15 Federal Institute of Hydrology, Department of Microbial Ecology, Am Mainzer Tor 1, 56068 Koblenz, Germany; 16 Faculty of Environment, Science and Economy, University of Exeter, Exeter, Devon EX4 4SB, UK; 17 Met Office, Exeter Devon EX1 3PB, UK; 18 Department of Political Science, University of Oslo, Oslo, Østlandet 0317, Norway; 19 Schweizerisches Epilepsie Zentrum, Klinik Lengg, Zürich 8008, Switzerland; 20 Geophysical Institute, University of Bergen, Bergen, Vestland 5007, Norway; 21 Department of Integrative Biology, University of Guelph, Guelph, ON N1G 2W1, Canada; 22 Department of Natural History, Norwegian University of Science and Technology, Trondheim, Trøndelag 7491, Norway; 23 Nuffield Department of Primary Care Health Sciences, University of Oxford, Oxford, Oxfordshire OX2 6GG, UK; 24 Department of Public Health and Nursing, Norwegian University of Science and Technology, Trondheim, Trøndelag 7030, Norway; 25 Clinical Research Unit Central Norway, St Olavs University Hospital, Trondheim, Trøndelag 7030, Norway; 26 Oxford Institute for Digital Health, Nuffield Department of Primary Care Health Sciences, University of Oxford, Oxford, Oxfordshire OX2 6GG, UK; 27 Norwegian Labour Inspectorate Authority, Trondheim, Trøndelag 7012, Norway

**Keywords:** Statistical physics

## Abstract

Quantifying uncertainty associated with our models is the only way we can express how much we know about any phenomenon. Incomplete consideration of model-based uncertainties can lead to overstated conclusions with real-world impacts in diverse spheres, including conservation, epidemiology, climate science, and policy. Despite these potentially damaging consequences, we still know little about how different fields quantify and report uncertainty. We introduce the “sources of uncertainty” framework, using it to conduct a systematic audit of model-related uncertainty quantification from seven scientific fields, spanning the biological, physical, and political sciences. Our interdisciplinary audit shows no field fully considers all possible sources of uncertainty, but each has its own best practices alongside shared outstanding challenges. We make ten easy-to-implement recommendations to improve the consistency, completeness, and clarity of reporting on model-related uncertainty. These recommendations serve as a guide to best practices across scientific fields and expand our toolbox for high-quality research.

## Introduction

Uncertainty is a well-acknowledged, fundamental part of the scientific process.^1^^,^^2^^,^^3^^,^^4^^,^^5^ Uncertainty in scientific work can take myriad forms and is generated from a wide variety of sources. No universal taxonomy of uncertainty exists,^6^ despite many efforts to classify and categorize the diverse sources and forms of scientific uncertainty.^3^^,^^7^^,^^8^^,^^9^^,^^10^^,^^11^^,^^12^ Generally, these taxonomies of uncertainty encompass three broad categories; uncertainty from natural randomness or variability in a system or process (aleatoric uncertainty), uncertainty in our knowledge of a system (including but not limited to; uncertainty in model structure, measurement and sampling errors, uncertainty in values of parameters), and uncertainty in our language, communication, and interpretation of processes. All of these sources are important contributors to scientific uncertainty, however, they cannot all be either quantified or reduced. In this article, we focus on the second category, uncertainty in a system or process, refining further to concentrate on quantifiable uncertainty associated with the use of statistical or mathematical models (model-related uncertainty).

The importance of uncertainty associated with the results of statistical and mathematical models is increasingly recognized because of prominent work in fields such as Climate Change^3^^,^^13^ and Epidemiology.^14^^,^^15^^,^^16^^,^^17^ Nevertheless, quantification of model-related uncertainty and its reporting is not consistent or complete^2^^,^^16^ within^3^^,^^5^^,^^16^^,^^18^ or between scientific fields.^1^^,^^19^^,^^20^ Despite similarities in descriptions of model-related uncertainty,^3^^,^^8^^,^^12^^,^^21^ a fully coherent picture has not emerged and different papers use different taxonomies of uncertainty and focus on different sources. There have been several calls for more consideration of uncertainty from specific fields or pairs of fields.^1^^,^^3^^,^^5^^,^^20^ But these have yet to be answered comprehensively. Other fields, such as Engineering and Meteorology, have well-established practices to deal with uncertainty. There are also some cross-disciplinary standards for specific uncertainty types, for example, measurement uncertainty through the International Standards Organisation,^42^ but even these standards can be too specific to be broadly applicable for cases of complex models.^12^ This inconsistency can lead to confusion as to the true level of uncertainty in results and hinder interpretability across fields.

With quantitative science now highly influential in the public sphere^3^ and the results from models translating into action, we must support our conclusions with sufficient rigor. Incomplete consideration of model uncertainties can lead to false conclusions with real-world impacts and an erosion of public trust in science.^16^^,^^18^^,^^22^ In 2019, Seibold et al.^23^ reported substantial declines in insect populations in Germany. This finding was widely publicized as an “insect Armageddon.”^24^ However, recent work by Daskalova et al.^18^ showed that a failure to account for uncertainty in model structure inflated confidence in the estimated declines. Only one of the five reported arthropod declines remained clear after uncertainty was corrected.^18^ In 2020, epidemiological models were at the forefront of strategies related to COVID-19. An over-reliance on communicating point estimates/predictions masked the full range of possible outcomes and potentially contributed to rushed, delayed, and inappropriate policy decisions and government action.^16^^,^^17^^,^^22^ Indeed, inquiries into the science of COVID-19 have now begun, with scientific papers considering the role of mathematical models used for policy decisions.^25^

All potential sources of uncertainty should be considered and accounted for when constructing, running, and interpreting statistical and mathematical models. The framework we use for our audit breaks model-related uncertainty into three primary sources: data (both observed and simulated), parameters, and model structure. The data element is further split into two sub-sources: the response, i.e. the focal variable trying to be explained, and the explanatory variables, i.e. any variables used to explain the response. This gives four sources in total to assess. An example of the framework as applied to a simple linear regression is given in Box 1. This “source framework” is broad enough to be applicable to multiple scientific fields, while still capturing the main sources of model-related uncertainty.

Box 1 **Example of source framework**

Focal model: a simple linear regression of change in height of plants as a function of temperature.

$$\;\text{Model}\;\text{equation:}\;\Delta H_{i}=\beta_{0}+\beta_{1}Temp+\varepsilon_{i}$$

$$\varepsilon_{i}=N\left(0,\sigma^{2}\right)$$

**Table:** 

Source	Element in the focal model	Example of potential uncertainty
Response variable	Change in height (ΔH)	Measurement/observation error
Explanatory variable	Temperature (Temp)	Measurement/observation error
Parameter estimates	Estimates of: Intercept (${\hat{\beta }}_{0}$), slope of relationship (${\hat{\beta }}_{1}$), and variance of the error (${\hat{\sigma }}^{2}$)	SE/confidence interval
Model structure	The structure of the equation	Comparison of alternative formulations e.g. non-linear structure or additional explanatory variables

Previous work has suggested that our current consideration of model-related uncertainty in the sciences is not sufficient,^1^^,^^2^^,^^3^^,^^5^^,^^18^ but the actual state of quantification and reporting in publications has not been assessed. To address this, we take a snapshot of the state of model-related uncertainty reporting from papers published at the end of 2019 across seven scientific fields (papers assessed: N = 545, papers remaining in analysis: N = 66 for Climate Science, 91 for Ecology, 56 for Evolution, 34 for Health Science, 89 for Neuroscience, 58 for Oceanography, and 93 for Political Science, Total = 480) to evaluate how they quantify and report model uncertainty in the key sources outlined above. These fields were chosen to represent a range of scientific disciplines that span broad subject areas (biological, physical, and one social science) but all have applied outcomes. Final field choice was determined by the collaborative network available to the lead author and those authors that had time to complete the systematic audit. Papers for each field were chosen by taking all original research papers from two field representative journals per discipline from the end of 2019 (further details are available in the STAR Methods in the supplementary files).

## How well are we currently doing?

**Figure 1:**
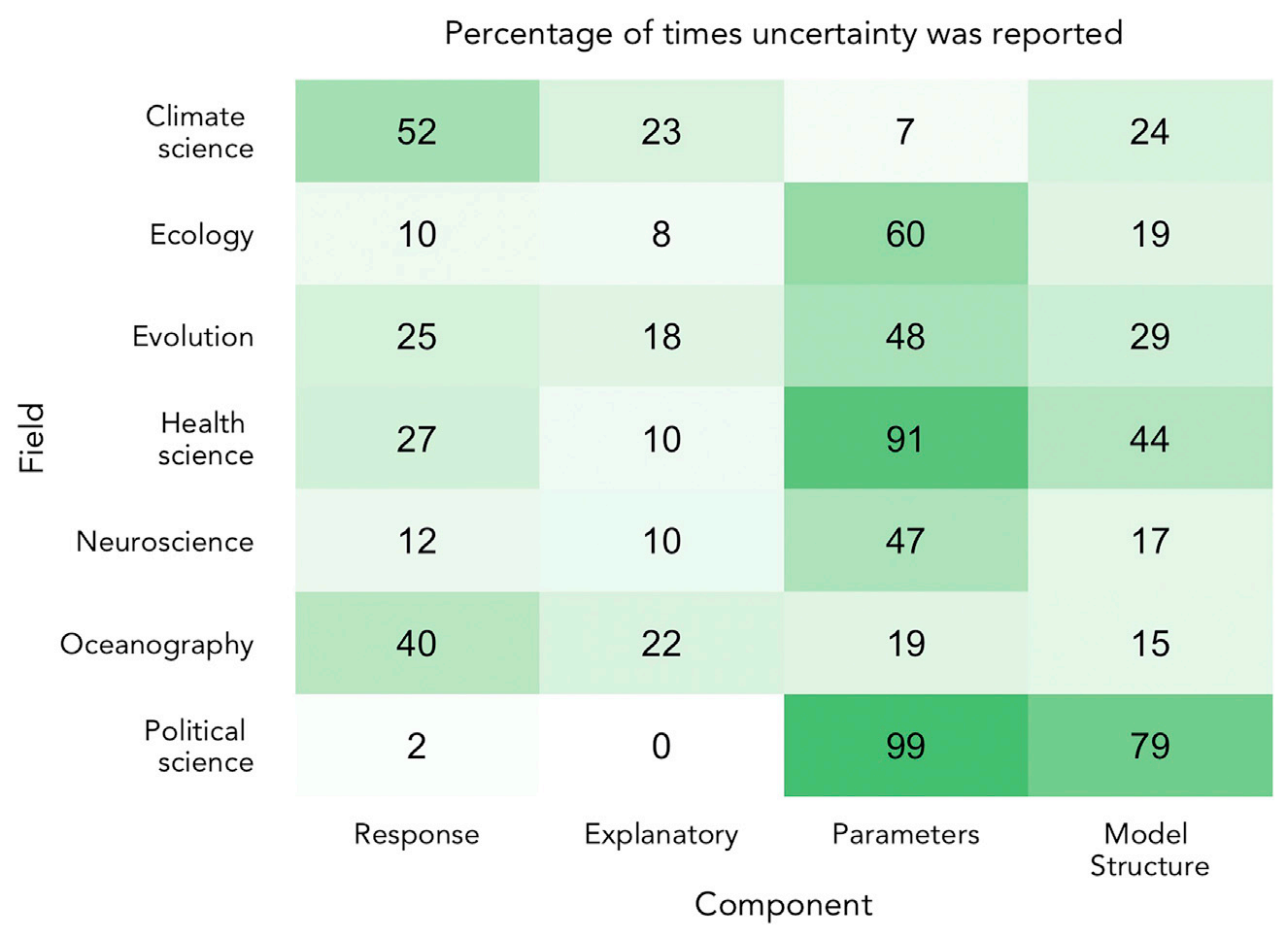
Heatmap of the percentage of papers that report uncertainty from each source split by field Positive results include papers that quantified and reported uncertainty arising from each source, when uncertainty was applicable. Cases, where no uncertainty was present in a source, were removed. Only one focal model was considered per paper assessed. (N = 66 for Climate Science, 89 for Ecology, 55 for Evolution, 33 for Health Science, 88 for Neuroscience, 38 for Oceanography, and 81 for Political Science).

The results of our snapshot assessment show that no field currently has a complete and consistent consideration of their model uncertainties (see Figure 1). However, across fields we get much closer to achieving this, offering opportunities for improvement; all four sources of uncertainty are quantified in at least 20% of instances within at least one field, with three sources having 50% or greater quantification in at least one field. Fields with low reporting of particular sources of uncertainty can learn from fields with high reporting of those sources. The one area where all fields fail to quantify uncertainty the majority of the time is from explanatory variables. The fields that perform best here are Oceanography and Climate Science, each reporting uncertainty in just under one-quarter of papers assessed.

**Figure 2:**
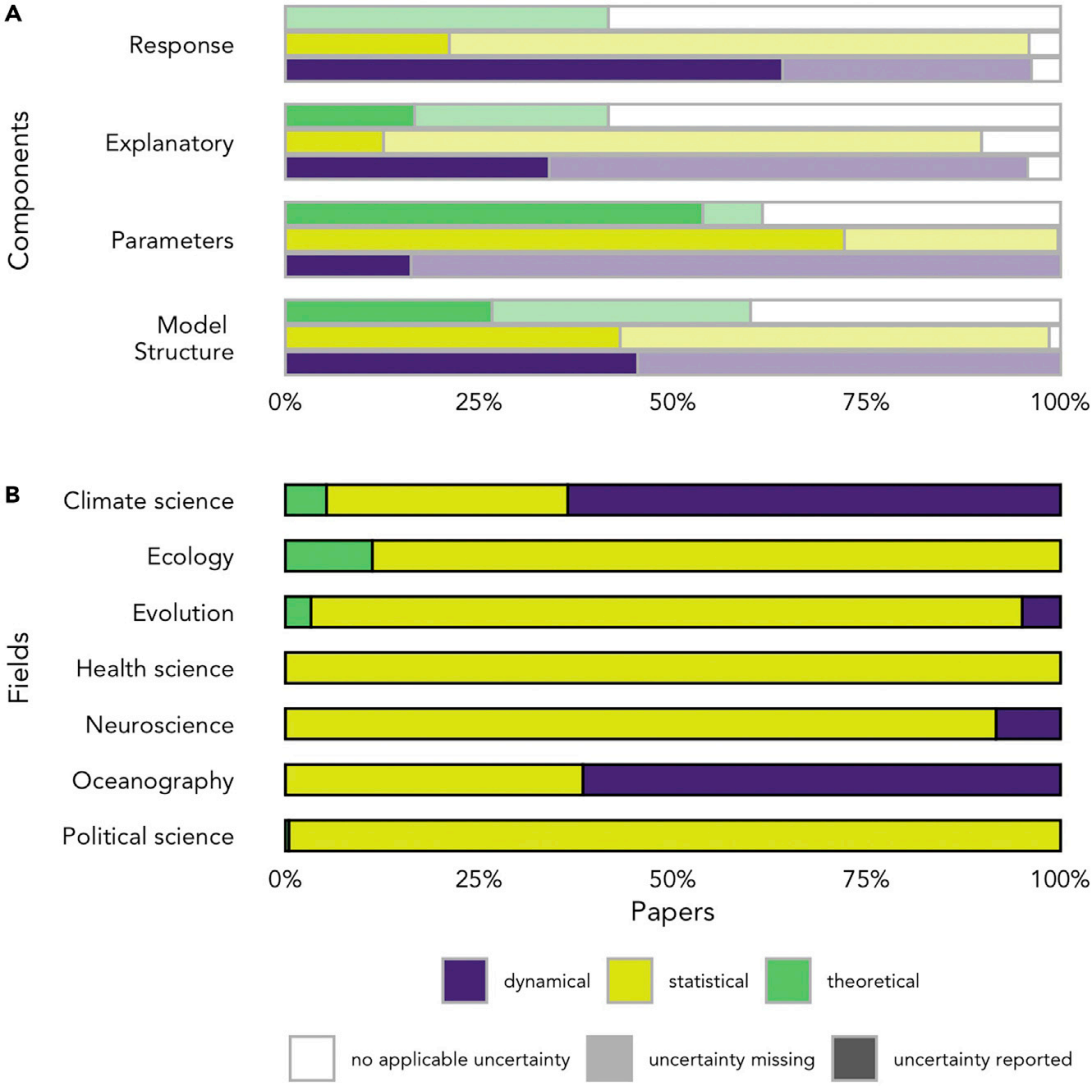
Distribution of different model types by uncertainty component and field (A) Presentation of the percentage of models in which there was uncertainty reported, uncertainty missing, or no applicable uncertainty for each source component (uncertainty was deemed not applicable if either the component was not relevant to the model or if there was no uncertainty in that component). (N = 57 for dynamical models (including mechanistic), 241 for statistical models, 12 for theoretical models or hybrid statistical/theoretical models). (B) Percentage of model type assessed by field.

We note that not all lack of quantification or reporting of uncertainty in a particular source represents an omission. There are some cases where quantifying uncertainty from a particular source is implausible, impractical, or unnecessary. Quantifying the uncertainty associated with a particular source may be implausible when the existence of error/bias/missing variables is totally unknown, or if this would necessitate investigation of all possible permutations of model. Examples of impracticality include huge models that would take an unfeasibly long time to run with quantified uncertainty and would be too complex to interpret meaningfully, or when modeling the uncertainty would require many assumptions due to a lack of knowledge about the true form of bias or error in the data. This is not uncommonly seen in some social science fields, where ambiguous data may mean that it is not possible to know if data are truthful.^26^^,^^27^^,^^28^ In this case, uncertainty is known to be present in the input data of a model, but the exact form is totally unknown and therefore cannot be practically modeled without multiple assumptions. Ultimately, these assumptions may be more subjective and add greater uncertainty than analyzing the data at hand. In these instances, discussion of the possible uncertainties and open acknowledgment of the limitations of the model to address them would be necessary as detailed in our good practice guidelines in Box 2. One example of a nuanced requirement to explicitly quantify uncertainty is for response data in statistical models. Commonly applied statistical methods based on linear models, such as linear regression and ANOVA, do account for uncertainty in the response when estimating uncertainty in parameter estimates. However, they do not report it explicitly. Generally, this lack of reporting does not matter and would not influence results because it is the relationship between the explanatory variables and the response that is of interest scientifically. In other situations, it is necessary to explicitly quantify uncertainty in the response of statistical models. For example, the survival of wild animals is typically derived from capture-recapture data, and its proper estimation requires explicit estimation of both the recapture process (observation) and a survival process. There are also cases where no applicable uncertainty is introduced from particular sources (see Figure 2). For example, in experimental studies, the explanatory variable is often a treatment group. These treatment groups are rarely a source of uncertainty in the modeling process, as group membership and treatment conditions are often known with certainty. Large numbers of experimental studies like this are present in Evolution and Health Sciences, and increasingly also in Political Science. A final example to note is when explanatory variables with noise or measurement error are actually the variable you want to represent such as in cases where explanatory variables are used for diagnosis or prognosis. In this case, it is the observed values of the explanatory variable which will be used for clinical use rather than the “true” values and representing the uncertainty between observations and true values would be unnecessary.

Box 2 **Good practice guidelines for all models types including case study implementation for each uncertainty source as identified during our assessment for statistical models**

Good practice common to all model types:

• Compare, contrast, or represent (through averaging of parameter estimates) the results of alternative model structures
• Discuss any uncertainty sources that were not quantified explicitly in the main text or discussion section and, explain why and detail how this could impact the results and conclusions of the analysis. Include a dedicated “uncertainty” section of the paper (possibly in supplemental information)
• Publish code and/or data/model output used for the analyses to ensure work is reproducible and reusable

Statistical model good practice:

• Quantify any error in the response data. This can be in the form of explicit modeling of measurement/observation error and subsequent correction, correction for non-independence, or an estimation of the error/bias that is propagated into the focal model
• Quantify any error in the explanatory variable data. This can be in the form of explicit modeling of measurement/observation error (using existing standards, e.g. those from the International Standards Organisation,^42^ where possible) and subsequent correction, correction for non-independence or confounding variables, an ensemble approach to represent multiple data sources, or an estimation of the error/bias that is propagated into the focal model. If explanatory variable data comes from another model output (as is the case for projections of future climate) the full uncertainty associated with this output should be propagated into the focal model
• Present error estimates or an interval representing the plausible parameter space for all unknown parameters. This could be as a confidence interval, credible interval, bootstrap interval, or standard errors.

Dynamical model good practice:

• Quantify any uncertainty entering the model from the response, if necessary (i.e. when the response is not a predicted outcome of the model and the aim of the model is quantitative understanding). This can be in the form of reporting model parameters (e.g. probability of detection) or statistics such as repeatability
• Quantify any relevant uncertainty in the explanatory variable data. This can be realized by running model ensembles with perturbed fields of the explanatory variables (i.e model forcing or initial conditions in most cases) with the strongest influence on the studied response
• While assessing uncertainty in all parameters in a dynamical model can be unfeasible (especially for data-intensive modeling such as climate science or oceanography), parameter uncertainty can be quantified similarly to that of explanatory variables by running model ensembles covering a range of possible values for the parameters in those equations with the strongest influence on the studied response
• In addition, for both uncertainties in explanatory variables and parameters, simplified versions of the models could be used for more comprehensive uncertainty analysis. Although the uncertainty quantified via such an approach would not be identical to that of the original more complex model, it would provide a dependable estimate

Theoretical model good practice:

• Check if it is necessary to quantify uncertainty in the response. Quantify any uncertainty in the response data. Typically, a response in a theoretical model will be the outcome of the model rather than an input and is therefore predicted. The response in this case is not strictly a source of uncertainty but it does accumulate uncertainty from all other sources. Therefore, to correctly represent uncertainty in the response, it is necessary to present the response accounting for the uncertainty introduced from all other sources. This can be in the form of presenting intervals around predicted response values, presenting a distribution of response values, or a range or other summary statistics that include variability of the results. May not have uncertainty if it is a deterministic model.
• Quantify any relevant uncertainty in the explanatory variable data. Explanatory variable data can either come from observations, experiments, or be simulated as part of the model. Each form of the explanatory variable is a source of uncertainty in a different way. This uncertainty can be quantified using measurement/observation error modeling, choosing a range of values/sampling values from a distribution during a simulation or bootstrapping, or sensitivity analyses to assess the impact of changes in explanatory variable values.
• Parameter values in theoretical models are often chosen *a priori* or optimized using various algorithms. Often parameters are chosen specifically based on previous scientific findings, from observed data, from knowledge of physical or chemical processes, or to test a specific theory. Uncertainty in these parameters should be quantified by choosing a range of values/sampling values from a distribution during a simulation or bootstrapping or sensitivity analyses to assess the impact of changes in explanatory variable values. In some cases, there will be no uncertainty added from the unknown parameters because the question being asked is dependent on specific parameter values, for example, does an increase of 1°C in mean sea surface temperatures cause greater carbon drawdown into food webs? In this case, the temperature parameters would need to be fixed and therefore would not be a source of uncertainty.
• Compare, contrast, or represent (through averaging of parameter estimates) the results of alternative model structures. This is not always relevant in theoretical models when they test if a specific model structure can produce the outcome expected. This is because the aim of many theoretical models is to test the model structure specifically. However, in other cases, where the motivation is simply to find a model that can represent reality, infinite different options could be available and some consideration of this breadth should be included.

**Table:** 

Cross-disciplinary example implementations for statistical models:			
Field	Source of uncertainty and quantification metric	Citation	Details
Evolution	**Response variable:** Measurement error	Conith et al.^43^	Response: Conith et al.^43^ conducted a comparative analysis of the evolutionary history of snout length and depth for cichlid fish. They corrected their response variables of snout length and depth using a theoretically expected variance-covariance matrix of correlation among traits which controls for non-independence from shared evolutionary history and taking account of the body depth of each specimen. This produced a “corrected” response variable that was independent of size and history, thus resolving uncertainty in what morphological or evolutionary processes this variable represents. **example of correction for non-independence**
Oceanography	**Explanatory variable:** Measurement error	Saderne et al.^44^	Explanatory: Saderne et al.^44^ used a mechanistic model to explore differences in the CO_2_ system across three ecosystems (coral reefs, mangroves, and seagrass meadows). One explanatory variable was pH on the total scale. This variable was corrected using extra data collected using a different method and the corrected variable was used in further analyses. Other input data elements (which were used as parameters in the final model) had their errors propagated using the R package *Seacarb*.^45^ This allowed for instrument errors to be accounted for throughout the analysis. These techniques for considering and accounting for errors in explanatory variables can and should be applied across statistical models as well. **example of correction for measurement/observation error**
Political Science	**Parameter estimates:** Interval	O’Grady^46^	Parameters: O’Grady^46^ modeled preferences for increases in federal social spending with explanatory variables of household income and subjective assessment of unemployment risk. The model used was a multivariate regression. Uncertainty in the parameter estimates from this regression was calculated using standard errors and presented as clustered standard errors numerically in Table 1^46^ and then as 95% confidence intervals around the coefficient estimate visually in Figure 5.^46^ **example of error estimates using standard errors**
Climate Science	**Model structure:** Model comparison	Fan et al.^47^	Model structure: Fan et al.^47^ compared two different parameterizations of a dynamical model for solar energy distribution and surface hydrology over the Tibetan Plateau. The dynamical model was the Community Climate System Model (CCSM4). One parameterization used 3D radiative transfer and the other used plane-parallel radiative transfer. The results of both parameterizations were compared visually in figures, as text, and numerically. Consideration of different processes and how they could impact results should also be considered in statistical models. **example of comparing results using two model structures**

Which sources of uncertainty were quantified and reported varied between fields but also by model type (see Figure 2). We classified all models in the audited papers into three broad model types: dynamical (a mathematical model based on the fundamental understanding of natural processes such as physical or biochemical laws), statistical (a mathematical model that represents a data generation process, e.g. linear regression), and theoretical (a mathematical model designed to illustrate or test a theoretical idea, typically does not include observed data). Variation was found in how often uncertainty was quantified as well as whether any uncertainty was applicable for each source across the model types (Figure 2A). While most sources in our framework could contribute uncertainty to the majority of models, theoretical models (including hybrid statistical/theoretical models) were more likely to have source components with no applicable uncertainty. The differences in uncertainty relevance and quantification by model type align partially with the boundaries of the scientific fields considered (see Figure 2B) as some fields focus more on dynamical and mechanistic models (Climate Science and Oceanography), while others rely more on statistical models (Health Science, Neuroscience, and Political Science), and some fields are more mixed (Ecology and Evolution).

We suggest that differences in uncertainty quantification are driven by the differing perceived importance of the sources of uncertainty for each model type and for specific research questions, as well as by practical considerations. For example, parameter estimate uncertainty receives the most consistent acknowledgment for statistical models. This is likely because a fundamental aim of statistical analyses and tools is to estimate and draw inferences from unknown parameters, and common standards exist for quantifying their uncertainty. In contrast, the aim of dynamical models is often to predict a response. In this case, uncertainty in the response was quantified most consistently, representing its greater focal importance for this model type in addition to the impracticality of quantifying uncertainty in the huge numbers of parameters and explanatory variables in complex geoscientific models. Additional gaps in uncertainty quantification are driven by the lack of tools or guidelines for quantification associated with particular model types and by author omissions and trade-offs. We present below some opportunities to improve our practice based on our interdisciplinary insight into these challenges.

## Cross-disciplinary collaboration highlights opportunities for improvement

Working with a large interdisciplinary team and informed by the results of our assessment of current practices, we identified several opportunities for improvement, summarised in Table 1.

**Table 1:** Table of identified opportunities to improve uncertainty quantification and reporting, including details of the improvement

Identified opportunity	Detail	Exemplary fields	Fields that can benefit
Greater consistency	Use overarching source framework to identify potential routes for uncertainty to enter models, then follow model-type-specific guidelines of good practice for quantifying and considering these sources	For statistical models: Ecology, Evolution, Health Science, Neuroscience, Political Science For dynamical models: Climate Science, Oceanography For theoretical models: Ecology	Fields that use multiple model types: Climate Science, Ecology, Oceanography
			Fields that are not yet as consistent in reporting: All
More complete uncertainty consideration	Share proposed good practice methods for quantifying uncertainty from different sources across model types and fields. See our guidelines and examples in Box 2.	Response: Climate Science, Oceanography Explanatory: Evolution, Oceanography Parameters: Health Science, Political Science Model Structure: Political Science	All fields
Effective presentation	Recommended minimum numeric presentation to aid reproducibility and reusability of results and reduce ambiguity. Also recommend combining with visual presentation when feasible to aid interpretation.	Strong visual presenters: Climate Science, Ecology, Evolution, Neuroscience Strong numeric presenters: Climate Science, Ecology, Oceanography, Political Science Strong across all: Health Science	All fields

### Greater consistency through a common framework

Achieving consistency in the quantification and reporting of model-related uncertainty across scientific fields is a challenging aim. Cross-discipline harmony has been hindered by both the lack of a standardized framework for considering model-related uncertainty and by field-specific vocabularies and different compositions of model types. Here we propose three complementary solutions which can help researchers address these challenges and produce easier cross-disciplinary comparisons. The first solution is a broad framework and common language through which to consider model-related uncertainty. We propose the use of our source framework as a tool to identify potential routes for uncertainty to enter the modeling process before referring to model-type-specific criteria for quantifying or addressing those uncertainties. The usability of this framework across different disciplines has already proven itself through our analysis. While we have demonstrated the usefulness of our framework across a broad range of fields, our coverage of social sciences was limited to Political Science. However, we are confident that the broad nature of our framework makes it applicable as a checklist for any quantitative model, regardless of discipline. For fields where a substantial part of uncertainty in a piece of work is not quantitative or quantifiable, other additional categories or standards may be required to further align uncertainty consideration beyond model-related uncertainty. The field of Engineering has already made substantial progress here, with specific methods available for the quantification of uncertainty in qualitative as well as quantitative data.^12^ While these additional elements are beyond the scope of this current work, we strongly encourage more research effort to create a harmonized approach to all types of uncertainty and expanding further into the social sciences in particular but retaining an interdisciplinary approach.

The second solution we propose is to follow cross-disciplinary good practice guidelines, which we present in Box 2. Our audit notes that differences in model uncertainty quantification seem to be driven by model type rather than purely by scientific field (see Figure 2B). As the sources of uncertainty in a particular model type are likely to be consistent across fields, we propose a logical split for guidelines of good practice by model type rather than the scientific field. By focusing on the model type and providing guidelines of good practice, as detailed in Box 2 for a statistical model, it is possible to achieve greater consistency and completeness in model-related uncertainty quantification across all scientific fields. Dialogue across fields is key to achieving greater consistency.

An example of the benefits of cross-disciplinary dialogue can be to see commonalities in our challenges. For instance, in some social sciences data ambiguity can be a challenge (in some cases it is not possible to know if data are correct/truthful^26^^,^^27^^,^^28^), while in fields such as Health Science, tests for diseases can give false positives or false negatives, and in fields like Ecology or Engineering, inaccuracy due to measurement or observer errors are common.^12^^,^^29^ While the causes of these issues are field specific (and even case specific), the impact they create for analysis and uncertainty quantification is shared. Both ambiguity and inaccuracy lead to incorrect observations of the process of interest. By highlighting such commonalities, it is possible to share solutions rather than the duplicating effort.

### More complete uncertainty consideration

Across all the considered fields, we have documented the quantification of four sources of model uncertainty. However, no field or model type alone achieves this. Our proposed good practice guidelines (with specific examples in Box 2), informed by cross-field examples and practices, can support more complete uncertainty consideration for all models used across the sciences. For each potential source of uncertainty for each model type, we give indicate how uncertainty could be quantified, which in turn could be adapted to the model at hand.

The guidelines in Box 2 leverage good practice from fields with specialized modeling repertoires to create a comprehensive set of uncertainty practices that is relevant to all fields, particularly those using a diverse modeling repertoire. An example of good practice from specialized fields is the quantification of uncertainty from parameter estimation in Political and Health Science. Both fields have standard presentation styles for this type of uncertainty, which is most commonly associated with some form of regression analysis. In Political Science, standard practice is to report standard errors (or occasionally t-values) for regression coefficients. It is also common to visualize uncertainty by plotting coefficients or marginal effects with 95% confidence intervals. The numerical and or visual presentation of 95% confidence intervals is standard practice in Health Sciences. These accepted standards have led to an almost 100% success rate in reporting uncertainty from parameter estimates in the papers we assessed for Political and Health Sciences (see Figure 1). In contrast, in Ecology, despite also using predominantly statistical models, there is no such universal standard. Ecology research employs many different modeling tools and software platforms or packages, including a number of user-defined models. This lack of consistent standard results in only a 60% reporting rate of parameter uncertainty in Ecology. Implementing clearer minimum expectations from statistical models like in Political or Health Science (as detailed in Box 2) could improve the quantification of parameter uncertainty in Ecology and other fields. We have based our recommended methods on currently available tools and good practice from the seven considered fields, but we see these as working guidelines that should be updated as new tools become available.

One area that should be addressed in greater detail is how to deal with uncertainty in a particular source when quantification is implausible, impractical, or unnecessary. This frequently occurs and can arise for many reasons including the complexity of the model, logistical constraints, lack of knowledge of the form of uncertainty, or methodological constraints. In these situations, applying our framework could be a useful method of systematically identifying what potential sources of uncertainty are missing from the current model, whether they could be incorporated or if they are quantified elsewhere. Researchers should subsequently consider how failure to quantify uncertainty is likely to affect the study conclusions, this approach could be a useful addition to the inquiry into COVID-19 modeling. This can include a detailed discussion of uncertainty elements that are missing from the quantitative assessment. This approach would be especially useful for fields that have a large component of uncertainty that is not quantitative or quantifiable, such as those with a large qualitative component. However, it should be noted that some methods to quantify qualitative uncertainty do exist.^12^ It also allows a broadening of the uncertainty consideration beyond that which can be easily given a numerical value.

Emphasis should be put on quality over quantity when applying these good practice guidelines. In theory, it could be possible to tick all boxes of addressing uncertainty from the four sources, without ever quantifying them correctly or thoroughly. The quality of the methods used to quantify uncertainty or how those methods were reported is not something we addressed in our assessment of papers; however, it is something that should be considered in future assessments and when developing good practices.

### Effective presentation of uncertainty

**Figure 3:**
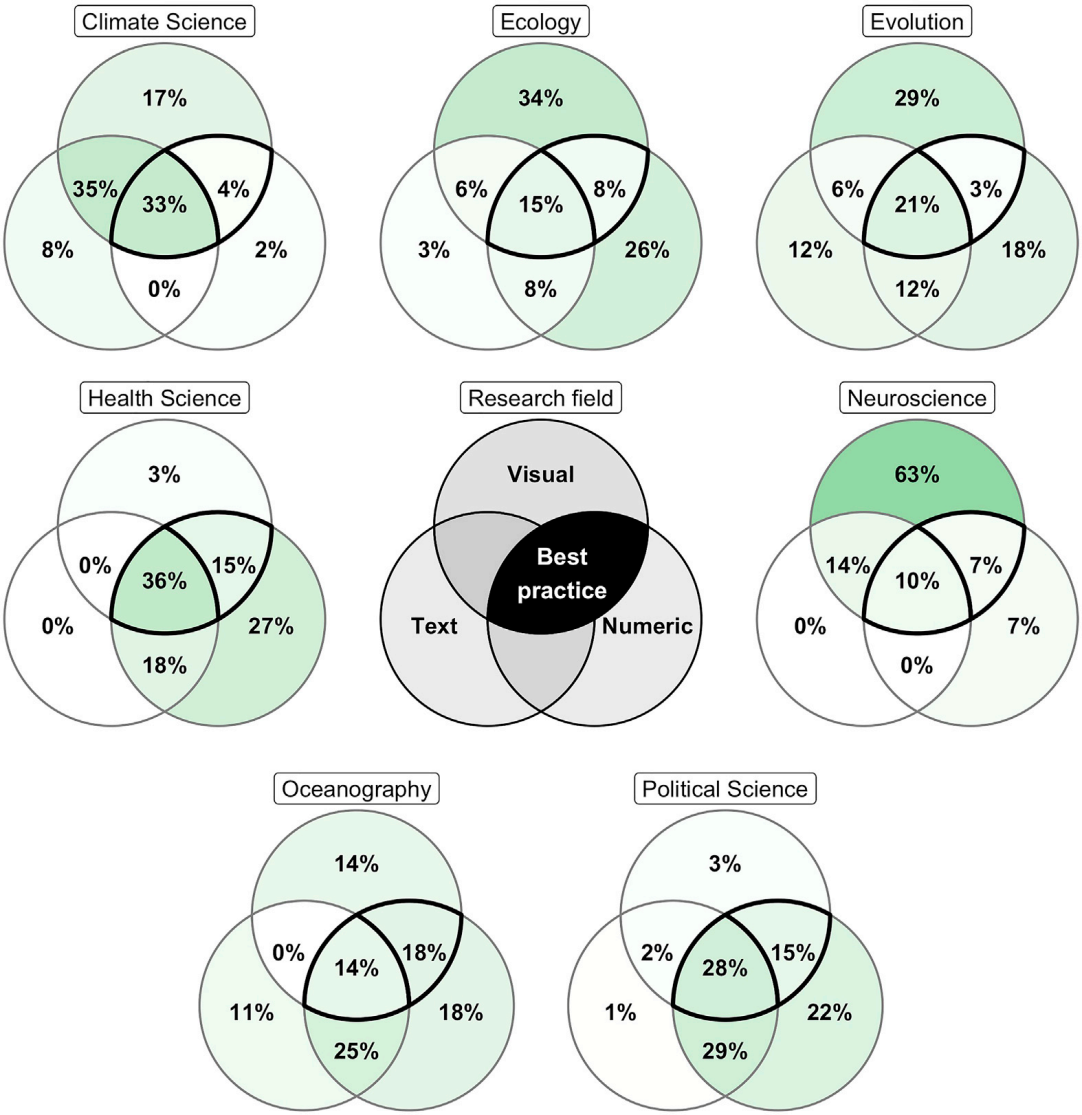
Venn diagrams of presentation methods for uncertainty (legend in the center) by field Black edged segments are our good practice recommendation of visual + numeric or visual + numeric + text.

It is not sufficient to only quantify model-based uncertainty; it is also essential to communicate it. Model-based uncertainty can be communicated in three primary ways: numerically, visually, or narratively. Across fields, different combinations of these communication types were used (see Figure 3), ranging from 63% visual-only communication in Neuroscience to >90% communication including numeric values for Health and Political Sciences to a balanced use of all communication types individually and in combination in Evolution and Oceanography.

While there exists a wide literature base discussing the most effective ways to communicate uncertainty, these papers often focus on a non-academic audience of policy makers or the public.^30^^,^^31^^,^^32^^,^^33^^,^^34^ Their findings suggest that openly communicating bounded or quantified uncertainty can increase trust in results^32^^,^^33^^,^^35^ and numeric and/or visual communication are more precise and effective than the textual presentation for communicating the desired uncertainty level.^2^^,^^31^^,^^36^ Several findings from this existing literature can also be useful for scientists when they are communicating uncertainty to an academic audience. We propose a minimum presentation of quantified uncertainty as numeric values in scientific papers, either in the main text, in the supplemental information; or in a supporting dataset published along with the paper. We recommend numeric presentation for two reasons, first, to reduce the ambiguity that could come from textual or visual presentation^2^^,^^37^^,^^38^ and second, to aid in the reusability of results. Numeric presentation of uncertainty is essential for the reuse of results in systematic reviews or meta-analyses, or for reproducing the results in the future and therefore is essential for the progress of research. We also recommend including the visual presentation when feasible to aid interpretation.^2^^,^^31^^,^^39^ There are many ways in which uncertainty can be presented visually, increasing the potential for an effective method to be found.^2^^,^^38^ Presenting model results visually can aid with the understanding of complex relationships but are not free from bias or misinterpretation, which is why we recommend a combination of numeric and visual communication.^38^^,^^40^ We recommend as a best practice that the code used to produce uncertainty presentations is shared to enhance the replicability and transparency of uncertainty quantification.

To effectively implement our communication recommendations, we advise developing a set of standard uncertainty analyses and tools to implement them (either within existing software/packages or as post-processing steps) so that every modeler can generate uncertainty metrics for their work. This would allow easier production of visual or summary tabular representations of model-based uncertainties, which can be included in the main manuscript text. This should then be coupled with larger tables of numeric values in supplemental information including full uncertainty bounds for each quantifiable source from the source framework. Again, we can take inspiration on good practice for uncertainty communication from some of the fields included in our audit. For example, Health Science presents uncertainty using numeric, visual, and text methods 36% of the time and includes some numeric representation >95% of the time. An example of good practice we encountered was Heisser et al.^41^ who coupled visual presentation with numeric bounds in Figure 3.^41^ In contrast, other fields such as Climate Science and Neuroscience report uncertainty numerically less than 50% of the time. These fields can learn from standard practice and examples from Health Science to improve their own uncertainty communication. The transferability of good practice will depend somewhat on the model used, for example, the visual presentation of parameter uncertainty for a model with greater than 100 parameters will not be practical. However, inspiration can still be taken to improve the communication of uncertainty when the presentation is practical.

## Outstanding challenges

In addition to the opportunities for improvement identified in the section above, we also note shared outstanding challenges to effective and comprehensive quantification of model-based uncertainty that spans the included fields and model types. These challenges are yet to have satisfactory solutions for any field. We discuss each challenge in detail below and propose some next steps for the research community to create a path to overcome them.

### Input data uncertainty

Across all fields assessed here, the source of uncertainty reported least often is for the data sources. Uncertainty from explanatory variables was quantified and reported <25% of the time and uncertainty from the response was reported <52% of the time across fields (see Figure 1). The slightly higher reporting rate of uncertainty in the response is driven by greater reporting for dynamical models (see Figure 2). This is because responses in these models are rarely in the form of input data, instead being predictions or validation data. It is the quantification and reporting of uncertainty arising from input data (observed data used as inputs to a model) that we identified as a particular challenge, but the consequences of ignoring it can be severe.^48^ Uncertainty can enter the modeling process from input data (both the response and explanatory variables) through random noise, unknown measurement or observation errors, or missed processes that could lead to imprecise predictions. We identified three key challenges that are preventing the quantification of input data uncertainty. The first is logistics, as it is not always possible to obtain sufficient measurements to be able to quantify uncertainty in particular variables. This is more common when data are very costly or logistically challenging to collect. The second and third barriers are related and comprise both a lack of knowledge of methods to quantify input data uncertainty and a lack of knowledge of the impact of failing to quantify input data uncertainty in different situations.

Tools do exist to quantify or remove most of these sources of uncertainty. There is a long history of using various error-in-variables models to account for uncertainty in the measurement of explanatory variables^49^^,^^50^^,^^51^^,^^52^ and international standards do exist,^42^ although it is suggested these standards may not be applicable to highly complex models.^12^ These models are supported by detailed theory in relation to linear models^52^^,^^53^ and some non-linear models^49^^,^^54^ and there is a myriad of options of models to quantify this type of uncertainty or bias. Models also exist to quantify observation error in response and explanatory variables, through the mapping of observed data to an unobserved or latent state (state-space models), which are widely used in demographic ecology.^55^ Despite the availability of these tools, our results show that generally across all included fields, it is not standard practice to employ them. Wide-scale implementation of such methods has been hindered by the lack of knowledge of their practical applications, insufficient availability of data, no rules of thumb for when errors will influence results in many cases, and not including such considerations in standard statistical teaching. For response variables specifically, as mentioned in the introduction, many standard statistical models already adequately account for this uncertainty provided uncertainty in the response follows a normal distribution. This results in little reporting of uncertainty in the response for statistical models, which largely is not an omission. We would go so far as to not recommend any extra consideration for uncertainty in the response in these cases since it is adequately accounted for by the standard practice. However, it is still important to note that it is not always the case that these assumptions are met. In some situations, further consideration and explicit quantification may be required and yet omitted as it is not standard practice to consider uncertainty in the response when checking for deviations from the assumptions of the model.

We suggest a single solution to all three barriers to our consideration of uncertainty in input data. We call for further theoretical or simulation work exploring the impact of unquantified uncertainty in observed data in different contexts, especially when repeat data collection is challenging/impossible. This work should be coupled with better communication of the methods and easy platforms or packages to implement them. This final step would open accessibility to a wider range of scientists even without a formal statistical background.

### Model complexity/logistics (including machine learning and artificial intelligence)

Increased computing power, improvements in data collection technologies, and developments of machine learning and artificial intelligence (AI) have allowed us to develop more complex statistical models of hard to study systems and automatic algorithms to fit them. However, with these increases in complexity come trade-offs in terms of quantification of model-related uncertainty and in interpretability, with many of these complex models often being treated as a “black box.” In parallel, complex models are frequently required in Climate Science and Oceanography to model the highly complex Earth system. Such models are often the only insight we can have into the behavior of physical systems on earth and are the best we can achieve with current tools. Using these complex models is therefore a crucial step in the progress of science.

However, it can be impossible, due to practical limitations, to quantify uncertainty in all parameters or input data sources for highly complex models,^56^ for instance, the computing resources required to quantify sensitivities for each parameter in a highly complex model could be beyond what is currently achievable. In some cases, attempting to quantify uncertainty in all parameters can actually reduce the accuracy of the model, having the opposite effect on the intention.^56^ A trade-off can therefore be made in deciding how to play off increased complexity against the assessment of uncertainty. In many cases, being more thorough about uncertainty can mean doing worse in terms of accuracy—for example, increasing model resolution may resolve a new process (e.g. eddies in the ocean) which means the result jumps out of the region where an uncertainty analysis at lower resolution would have bounded the problem, but now falls close to where the real truth lies. Therefore, caution should be exercised when trying to address all sources of uncertainty in complex models and ensure we have the correct tools to achieve this successfully. The trade-off exists between what we can now model and what we can interpret and quantify uncertainty for.

We suggest two solutions to the trade-off between model complexity and uncertainty quantification. The first is to include a specific uncertainty section of all manuscripts potentially as a designated supplemental information section. This uncertainty section would contain the discussion of the limits and assumptions of the models in terms of uncertainty. A dedicated section would also give space to discuss the potential consequences of any unquantified uncertainty, including reductions in accuracy or giving indications of which elements could be expected to change. The second is to call for further research to improve methods for quantifying uncertainty in complex models and model fitting algorithms, ensuring uncertainty quantification keeps pace with model development.

### Propagation/within paper consistency

During our audit, we noted two issues with the propagation of model uncertainty. The first was within the models in a paper, where we observed that in some papers with multiple analyses, uncertainty consideration was not consistent across all models. This pattern was especially prominent for papers including multiple analysis types within a single study, a more common occurrence in fields such as Ecology, Evolution, and Neuroscience. The second was in the propagation of uncertainty in results into the final discussion and conclusions. Rarely did we find that quantified uncertainty was propagated through into the discussion and conclusion of manuscripts. This pattern was universal across all fields. Even when uncertainty was reported earlier in the manuscript, conclusions still were largely based on point estimates or mean patterns. Uncertainty that was mentioned in the discussion sometimes focused on missing processes or caveats to conclusions rather than quantified uncertainty. Both of these propagation issues can hinder the interpretation of the uncertainty associated with results.

We propose using our good practice guidelines for different model types to ensure consistency in uncertainty consideration across all models in papers. Using a source-based framework for these guidelines helps to identify where uncertainty enters the modeling process and therefore improves the propagation of uncertainty. We also encourage journals to set an expectation for paper conclusions and discussion to include references to model-based uncertainty. Currently, there can be trepidation among authors about diluting their conclusions by incorporating uncertainty, especially due to the highly competitive publishing and funding environment and the potential of public influence. Improving acceptance of transparent uncertainty in scientific conclusions would be a way forward to propagation into final results.

### Interdisciplinary working

It is increasingly recognized that an interdisciplinary approach is required to address many of the key questions facing society, including climate change, the biodiversity crisis, and global pandemics. Much of this work revolves around statistical or mathematical modeling, often integrating approaches from multiple disciplines. Quantification of uncertainty is essential for the effective application of this work to societal decision-making, but the lack of a common framework for understanding uncertainty across fields makes it difficult to assess uncertainty in complex multidisciplinary systems.^57^ We propose that our push for greater consistency in the quantification of uncertainty across fields will facilitate better reporting of uncertainty in interdisciplinary work, which we expect will aid interpretation and application by multiple audiences.

### How much uncertainty consideration is enough?

It is not possible to account for all possible uncertainty in our studies,^5^ there will always be unknown unknowns that remain and known sources that for practical and well-considered reasons cannot be addressed in a given study. However, we do need to ensure that we make best efforts to represent as much of the uncertainty related to our results as we can and in the correct way. How we can achieve accurate uncertainty representation without over complicating or diluting results, remains an open question. In relation to the framework we present, we need to ask: are there times when explicit the quantification of all sources is actually not enough? Model-based uncertainty is not the only uncertainty associated with scientific work.

How much is enough will be case-specific and nuanced. It does not only depend on the type of model used but also the aims and focus of the work, for example, a regression model of heart rate (response) as a function of age (explanatory) where the authors do not care about the distinction between “true” and “observed” heart rate would not require explicit the quantification of uncertainty in the response. In this example, the main focus of the paper is on the relationship between age and heart rate (either observed or true) there is a strong need to be very sure of relationship and the uncertainty in that parameter and this need is satisfied because uncertainty from the response is accounted for in the estimation of the parameter and its uncertainty. In contrast, there could be a case where authors want to make conclusions specifically for the “true” value, not the “observation” and make predictions for it; for example, if managing a population of endangered animals. Here it is essential to explicitly know how the observed counts map to true population values, therefore requiring explicit quantification of uncertainty in the response. It can be exceptionally challenging to tease apart these nuances from published papers because it is not always explicit how the authors view each model component or how generalizable they intend their work to be. Furthermore, the limits and assumptions of models used are frequently not discussed in sufficient detail.

We propose a solution to illuminate the aims and scope of different model-based analyses and encourage authors to discuss all uncertainty in their work, even those elements that are not quantified. This would be achieved by having a specific uncertainty section of all manuscripts, as also suggested to aid uncertainty reporting for complex models. This section could include specific author statements on which sources of uncertainty have been quantified and why, which elements are missing and what their impact might be, as well as the intended scope of the work and any limitations. This would help readers to appreciate the full uncertainty associated with the work and aid in the correct reuse, replication, or citing of any results.

Some fears exist in the academic community about being explicit about uncertainty in our work, assuming that public or policy audiences might lose trust in results. Indeed, in some situations communicating uncertainty can influence public perceptions negatively due to ambiguity aversion, such as with vaccine effectiveness.^34^ However, there have also been several findings indicating users of official statistics and members of the public can engage well with model-related uncertainty.^2^ Some have even demonstrated that a lack of transparency around uncertainty can erode public trust,^16^^,^^18^^,^^22^^,^^58^ while communicating technical uncertainty can have positive effects on credibility.^59^ Therefore, we should not shy away from reporting uncertainties associated with our work but instead ensure we communicate them as fully and transparently as possible and in an easy to interpret manner. We will never have full control over how our work will be communicated by the media, read by the public, or used by policy makers. However, to have any chance that the full nuances of our studies are considered, we must provide them. As a result, we must be clear but also careful with how we communicate uncertainties associated with our work. Increasing transparency and consistency of our uncertainty reporting could help improve public trust and aid policy maker decisions.^38^

As mentioned in the introduction, uncertainty can enter the scientific process from a myriad of sources, with model-associated uncertainty being just one. As a result, it would be possible to score perfectly using our proposed source framework, quantifying all sources of model-based uncertainty in some way, but still have results that are subject to large unquantified uncertainties. Furthermore, in our analyses, we made no judgment about whether a certain method of quantifying uncertainty was the most appropriate or what impact the quantification had on results; for example, whether results actually became less accurate in the pursuit of better-quantified precision. Quality of uncertainty quantification will also be a crucial element in determining whether enough has been done in any given analysis. Simply ticking all boxes is not sufficient, good practice for each must also be followed (see Box 2).^17^

## Recommended ways forward

Our analysis reveals a lack of consistency in uncertainty quantification within and between fields. The fact that some fields do successfully account for uncertainties for certain model types and sources while others do not indicate that it is disciplinary protocols or customs that have led us to this state. Our analysis also highlights the potential for improvement. To reveal these previously unnoticed patterns, we had to translate the discipline-specific terminology surrounding model development and uncertainty quantification into a common language.

We make ten concrete recommendations for current practice, future work, and general research recommendations. The first two categories are aimed at the modeling community and the third is aimed more broadly, including at scientific publications and funders.

Recommendations for standard practice in quantifying model uncertainty:

1. Use the source framework as a structured tool for considering model uncertainty. Where uncertainties from the sources can and should be quantified, do so. Where it is not feasible or practical to quantify a particular source of uncertainty, instead include a theoretical discussion and acknowledgment of the missing uncertainty, why it is missing, and consideration of what impact it may have on the results reported.
2. Follow our proposed interdisciplinary good practice guidelines for uncertainty quantification (see Box 2).
3. Present model uncertainty as clearly as possible using at minimum some numeric presentation to aid reuse and reduce ambiguity. Should be combined with visual presentation when feasible to aid interpretability.
4. Propagate model uncertainties into the conclusions drawn from the work.

Recommended future research priorities:

5. Develop tools and guidance on how to identify when uncertainty from input data is important.
6. Couple modeling advances such as increased complexity or AI and machine learning with further theoretical work on how to quantify and propagate the uncertainties associated with such methods.
7. Conduct further research into the influence and importance of the different sources of uncertainty for final results and conclusions across multiple modeling types and contexts.
8. Expand our uncertainty framework to include areas beyond quantitative models. Particularly, do this in the context of a broader range of social sciences to better capture their unique issues in relation to uncertainty.

General recommendations:

9. Where uncertainty cannot yet be quantified and its impact is not known, be transparent about these limitations, especially when drawing conclusions. Be accepting of conclusions that include explicit recognition of model uncertainty
10. Make it standard to have transparent and easy access to quantified model uncertainties in all manuscripts, e.g. through standard dedicated supplemental information sections

Recommendations 1, 2, 3, 4, and 9 can be implemented immediately but recommendations 5, 6, 7, 8, and 10 require long-term planning.

## Limitations of the study

Although we employed a systematic approach to our audit of papers, alongside training, calibration, and repeated consistency checks, there are likely to remain small between reviewer deviations in scoring of papers. Unfortunately, we could not audit all papers that we aimed to at the beginning of this project. Time constraints of reviewers, particularly influenced by the coronavirus pandemic, led to different numbers of papers being audited per field. This means our final coverage was not equal across the different fields in this study. However, as the results are summarised as percentages and still cover a meaningful number of papers per field (minimum 34), they remain comparable. As mentioned in the main manuscript, our study included only a single representative of social sciences (Political Science), we strongly encourage further work expanding the application of our framework to a wider array of social sciences and to uncertainty types beyond quantitative uncertainty. In addition, this current work could not assess the quality or appropriateness of the reported uncertainties or what the consequences of omissions are for reported results.

## Supplemental information

Document S1: STAR methods

Table S1. First iteration of data collection table

Table S2. Second iteration of data collection table

Table S3. Included papers

Table S4. Excluded papers
