# Supplementary material for: Insights into the quantification and reporting of model-related uncertainty across different disciplines
Source: iScience. 2022 Nov 5;25(12):105512. doi: 10.1016/j.isci.2022.105512 (PMC9712693; doi:10.1016/j.isci.2022.105512)
Supplement: Document S1: STAR methods [file mmc1.pdf]

iScience, Volume *25*

Supplemental information

Insights into the quantification and reporting of model-related uncertainty across different disciplines

Emily G. Simmonds, Kwaku Peprah Adjei, Christoffer Wold Andersen, Janne Cathrin Hetle Aspheim, Claudia Battistin, Nicola Bulso, Hannah M. Christensen, Benjamin Cretois, Ryan Cubero, Iván A. Davidovich, Lisa Dickel, Benjamin Dunn, Etienne Dunn-Sigouin, Karin Dyrstad, Sigurd Einum, Donata Giglio, Haakon Gjerløw, Amélie Godefroidt, Ricardo González-Gil, Soledad Gonzalo Cogno, Fabian Große, Paul Halloran, Mari F. Jensen, John James Kennedy, Peter Egge Langsæther, Jack H. Laverick, Debora Lederberger, Camille Li, Elizabeth G. Mandeville, Caitlin Mandeville, Espen Moe, Tobias Navarro Schröder, David Nunan, Jorge Sicacha-Parada, Melanie Rae Simpson, Emma Sofie Skarstein, Clemens Spensberger, Richard Stevens, Aneesh C. Subramanian, Lea Svendsen, Ole Magnus Theisen, Connor Watret, and Robert B. O'Hara

STAR methods for paper: # Insights into the quantification and reporting of model-related uncertainty across different disciplines

Lead contact

Further information and requests for resources and reagents should be directed to and will be fulfilled by the lead contact, Emily G. Simmonds (emilygsimmonds@gmail.com).

Materials availability

This study did not generate any new materials.

Data and code availability

Section 1:

All cleaned data used in this study have been deposited in a Zenodo repository and are publicly available as of the date of publication. DOI: 10.5281/zenodo.7257247.

Section 2:

All code used to create the figures for this study has been deposited in a Zenodo repository alongside the data. DOI: 10.5281/zenodo.7257247.

Section 3:

Any additional information required to reanalyze the data reported in this paper is available from the lead contact upon request.

Method Details

The framework

The framework used for this review is a source-based framework and was developed specifically for this study. It breaks model-related uncertainty into three primary sources: data (both observed and simulated), parameters, and model structure. The data element is further split into two sub-sources: the response, i.e. the focal variable trying to be explained, and the explanatory variables, i.e. any variables used to explain the response. This gives four sources in total to assess.

Table: Description of framework elements

SOURCE COMPONENT	DEFINITION	HOW IS IT A SOURCE OF UNCERTAINTY?
Response	The variable(s) of interest, the quantity(ies) we want to explain or predict. Could be something measured or something latent (unobserved) or simulated.	If the response is observed, it can have unknown error and biases. If the response is latent and therefore never observed, then it needs to be estimated. Responses can also be output from another model e.g. interpolated temperature. Predicted responses will have all uncertainty from predictive model.
Explanatory variables	Any variable that explains or predicts the response. Could be something measured or something latent (unobserved) or simulated or theoretical.	If the explanatory variables are observed, they can have unknown error and biases. If the covariates are latent and therefore never observed, then they need to be estimated. Covariates could also be output from another model e.g. interpolated temperature. Theoretical covariates could have many plausible values (e.g. initial conditions in a simulation).
Parameter estimates	Values given to unknown parameters in the model either through estimation, optimisation, or chosen.	Can arise from the statistical estimation of unknown parameter values, which gives the range of plausible parameter values generated from estimation. Can also arise from optimisation or selection of parameter values for a theoretical model.
Model structure	Uncertainty in the process being investigated – the structure of the equations that link the response, explanatory variables and parameters. Can be called 'model uncertainty' in some disciplines.	Different model structures contain different assumptions, parameters, and covariates. They therefore produce different results for the same response.

Models typically contain all of the components of the framework. Nevertheless, one or several may be missing or minimally important in specific cases. Together, the components can be considered a modelling unit. As model complexity increases, the number of units also increases, leading to more

layers of uncertainty (e.g. in a predictive model). However, we do not assess these layers specifically here.

Example of the framework in practice:

Focal model: a simple linear regression of change in height of plants as a function of temperature.

Model equation:

$$\Delta H_i = \beta_0 + \beta_1\, Temp_i + \varepsilon_i$$

$$\varepsilon_i \sim N(0, \sigma^2)$$

Table: Expanded example of source framework (from Box 1)

Source	Element in the focal model	Example of potential uncertainty	Example of quantification
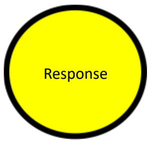	Change in height (ΔH_i), this element covers uncertainty in ΔH_i not $E(\Delta H)$	Unknown observation error	Explicit model of the observation process e.g. $$y_i = \beta_0 + \beta_1\, Temp_i + \varepsilon_i$$ $$\varepsilon_i \sim N(0, \sigma^2)$$ $$\Delta H_i \sim Pois(y_i)$$ Where y_i is the true change in height and ΔH_i is the observed change
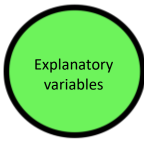	Temperature ($Temp_i$)	Unknown measurement error	Explicit estimation of the variation introduced to the explanatory variable by measurement error (σ_η^2) e.g. $$Temp_i^* = Temp_i + \eta_i$$ $$\eta_i \sim \mathrm{N}(0, \sigma_\eta^2)$$ Where $Temp_i^*$ is the true temperature and $Temp_i$ is the observed temperature
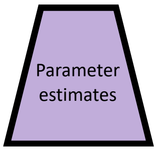	Estimates of: Intercept ($\hat{\beta}_0$), slope of relationship ($\hat{\beta}_1$), and variance of the error ($\hat{\sigma}^2$)	A range of values could be plausible as the 'true' parameter value for any parameter	Standard error/confidence interval
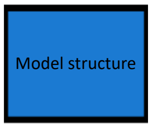	The structure of the equation	Other processes could influence change in plant height e.g. rainfall, or it could even be a non-linear relationship with temperature	Comparison of alternative formulations e.g. non-linear structure or additional explanatory variables

Model type definitions

While the term 'model' is often used as shorthand in specific disciplines to refer to the predominant model type, here we take a broader approach and identify three key types of model, which are defined below.

- *Statistical models***.** A mathematical model that represents a data generation process. These models aim to say something about a population of interest based on data from a sample taken from that population. Examples of statistical models are linear regression, t-test, analysis of variance.
- *Dynamical models (including mechanistic models).* A mathematical model based on fundamental understanding of natural processes (e.g. physical or biochemical laws). Dynamical models take the form of a series of equations based on physical and biochemical principles that are used to understand systems where there is change, growth, or development e.g. the climate system. They can be used to fit to or explain observed data or model output. Often applied to dynamical systems.
- *Theoretical models***.** Can be similar in motivation to dynamical models but contain no observed data (even in the form of model output to represent a real variable). These models are a theoretical exercise to test a particular idea. They are used to progress theory around certain processes.

Qualitative models were noted but excluded from this analysis as the framework is designed for quantitative models. Qualitative models were defined here as those models that are not quantitative (do not involve something that can be measured) but tried to give a conceptual understanding to a problem through specific experiences or behaviour. Qualitative models still try to draw inference about phenomena of interest and potentially assign causality, but they do so without a quantitative framework.

Development of Audit Strategy

Initial assessment criteria:

The first iteration of the data collection table (see Table S1 in supplementary files) was used to conduct an audit on 10 papers from the Journal of Animal Ecology from the September 2019 issue. The trial was conducted by all eight members of the Ecology team and all reviewers audited the same papers. The results of the trial were compared using percentage agreement (calculated as the number of reviewers with the same answer over the total number of reviewers). The consistency of results at this stage was low (often < 70% agreement). Agreement was particularly low for the section relating to an unobserved state.

The preliminary data collection table was split into two sections, one focused on an explanatory model and the second on predictions, if they occurred. Within each section there were two mini-tables, the first covered a state, or unobserved, process and the second covered the observed process. For each process questions were asked about what form each source component took and whether the associated uncertainty was reported.

Table: Summary of percentage agreement between reviewers in first trial of data capture table

**Component**	**Average percentage agreement**
Observed Response	48
Observed Explanatory variable	73
Observed Parameter estimates	37
Observed Model structure	50

Following the trial audit, all eight reviewers attended a three-hour workshop to give feedback on the table and discuss the inconsistencies. After the workshop, a new data collection table was drafted to streamline the data collection process and ensure results could be repeatable. During the initial trial audit, we found that the preliminary table had been too complex and with too high a focus on state vs observation processes. In streamlining the data collection table to focus more directly on each source of uncertainty and report more details on how uncertainty was presented, we ensured easier and more consistent data collection and a framework that was more easily applicable to other fields.

The revised data collection table is included as a supplementary file (see Table S2 in supplementary files). We reduced the number of questions down to nine numbered questions with seven answer columns. The first four questions covered the four sources of model-based uncertainty identified in our framework. Questions five and six covered whether there was an explicit model for an unobserved process, or state. Questions seven and eight covered whether predictions were made and if the associated uncertainty was reported. Question 9 assessed whether any quantified uncertainty was discussed in the discussion or conclusions. Each of the seven answer columns collected a different bit of information about the question, full details are shown in the instructions document below.

Refined assessment criteria:

A second trial, using the revised data collection table of questions (see Table S2 in supplementary files), was conducted on five papers from Ecology and Evolution from 2019 (this was a journal that was not included in the final audit to give an independent test of the method). This second trial was conducted by four of the original eight reviewers, due to time constraints of the other four.

Table: Summary of percentage agreement between reviewers in second trial of data capture table

Question number	Average percentage agreement
1	80
2	100
3	75
4	80
5	92
6	75
7	100
8	100
9	92

Agreement from the second trial was much higher (from 49% across all questions in trial 1 to 88% across all questions in trial 2) but not perfect, although absolute perfection is unlikely to be possible. The main continued areas of inconsistency arose from choosing No rather than NA for some sections. In response, a checks section was added to the spreadsheet for data entry for the final set of criteria. In addition, the detailed instructions document was refined and expanded and can be found below.

The Systematic Audit

Field selection:

The final fields included in these analyses were two biological sciences (ecology and evolution), two physical sciences (climate science and oceanography), two health/medical sciences (health science and neuroscience) and one social science (political science). These fields were chosen to represent a range of scientific disciplines that span broad subject areas (biological, physical, health, and one social science) but all have applied outcomes. Final field choice was determined by the collaborative network available to the lead author and those authors that had time to complete the systematic audit. A cross-disciplinary field of geography was intended to be included but time constraints of the collaborators meant this was not possible.

Search Strategy:

Unlike typical Systematic Reviews, we did not use search strings to target research articles relevant to our research question. Instead, we took **all** research articles from the last few issues from 2019 (the exact number depended on the number of research papers per issue and how often issues were released – the aim was for 30-50 papers per journal), of a population of academic journals across a broad-range of scientific disciplines. Our list of target scientific journals was built by consultation during workshops with the full audit team and had to meet the following criteria to be included:

- English language
- Some original research papers per issue
- International audience
- Field leading
- Frequently publish papers that use statistical models

While all original research papers from each issue of these journals were to be assessed, only those that included a statistical or mathematical analysis involving a model (it did not need to include observed data) were included in the audit.

Article types excluded from these analyses were determined by the following exclusion criteria:

- book reviews
- editorials
- in-focus pieces
- narrative reviews with no analyses
- no statistical, dynamical, or mathematical model
- purely descriptive work
- qualitative models
- meta-analyses and systematic reviews
- models on animals (Health sciences only)

Of the papers selected for the audit, 123 papers were excluded from the final assessment either due to the criteria above, due to time constraints of the reviewer, or because the assigned reviewer had to leave the project. The full table of included and excluded or not assessed papers can be found in the supplementary files (Tables S3 and S4).

The final list of journals used in the audit were:

Table: List of journals used in systematic audit

**Field**	**Journal**
Ecology	Journal of Animal Ecology
Ecology	Ecology
Molecular Ecology/Evolution	Molecular Ecology
Molecular Ecology/Evolution	Evolution
Climate Science	Journal of Climate
Climate Science	Climate Dynamics
Neuroscience	Nature Neuroscience
Neuroscience	The Journal of Neuroscience
Political Science*	American Journal of Political Science
Political Science*	British Journal of Political Science
Political Science*	International Organization
Health Sciences	British Medical Journal
Health Sciences	BMC Medicine
Oceanography	Journal of Geophysical Research: Oceans
Oceanography	Journal of Physical Oceanography

** Political Sciences assessed three journals in order to achieve a sufficient sample size due to a small number of original research articles per issue*

Extracting papers from the journals:

There were no fixed number of issues chosen for each journal, instead, enough issues were chosen to be able to extract around 50 original research papers per journal. This led to a total of 50-110 papers per field being extracted for audit.

Once research papers were extracted, they were randomly assigned to reviewers in each field. Each individual reviewer then screened their assigned papers for presence of a model and excluded any article types listed in the 'exclusion criteria'.

In total, we audited 50-110 papers per field. The exact number of papers audited was dependent on; time constraints of reviewers (this was particularly challenging due to increased pressures from the coronavirus pandemic), desired breadth of coverage of the field, and number of team members per field.

Reviewer training:

The first phase of the systematic audit was a reviewer training phase. This was conducted within each field team to ensure that all reviewers were familiar and comfortable with the instructions and framework, that all reviewers were applying the criteria consistently, and that the framework was applicable to papers from each field included in this audit.

The reviewer training phase consisted of all reviewers in each field auditing the **same four-five** papers. These papers were taken from one of the target journals for each field also from 2019 (but independent of the articles to be used for the main audit). The papers for the training phase were selected by choosing the first four-five papers in the target issue.

For Evolution/Molecular Ecology and Health Sciences the training phase was run slightly differently. Instead of the four-five papers being taken from a single journal two papers were selected from each of the two journals chosen for these fields. This tweak to the protocol was applied as the journals in these fields each have a substantially different style. In addition, the journal 'Molecular Ecology' has a sub-section structure, therefore, to ensure breadth of training papers, one paper was selected from the first subsection and one from second in the September issue of this journal. Both papers were the first papers listed in each sub-section.

Once all reviewers had completed the training audits their tables of results were sent to the project leader (E. G. Simmonds). The project leader then analysed the results by calculating percentage agreement scores in the same way as described above. A meeting was then held with each field team including the project leader to discuss the results of the training, answer any reviewer questions, address the causes of any low agreement, and ensure that the instructions were clear and easy to follow. The presence of the project lead in all of these field specific meetings ensured consistent interpretation and implementation of the audit framework across fields.

Where low agreement was noted during this training phase (low agreement defined as < 70% agreement), the causes of this were discussed in the summary meeting and a consensus achieved to resolve the cause of the inconsistency, to avoid discrepancies in future, and ensure between-reviewer consistency.

Following the first set of reviewer training (for the Ecology, Oceanography, Neuroscience, and Political Science teams), the instructions were updated to be more detailed and reflect the concerns raised during these meetings. This step ensured that any lack of clarity was immediately addressed and communicated to all members of the project, further improving between-reviewer consistency. Working documents of definitions of model types and uncertainty types were also created so if new model types or uncertainty types were encountered during the audit, these could be added in a way clearly visible to all reviewers.

The Health Sciences team chose to conduct a second round of reviewer training following the first to be sure of improved agreement.

Main audit phase:

The reviewers within each field audited their assigned papers using the data collection table and updated instructions. Each reviewer audited as many of their assigned papers as they were able within their time constraints.

A protocol was in place to deal with papers that any reviewer found challenging, or if they did not feel confident that their coding was repeatable.

Protocol for dealing with challenging papers (this was introduced in November 2020, in response to a need identified by the Ecology team and was implemented for all teams):

If during the audit any reviewer finds a paper challenging where they are unsure of answers, the following protocol will be initiated:

- The reviewer will send the paper to the contact person for their team and it will be distributed to other members (min of one but up to three) of their field team to assess if they agree with the coding by the focal reviewer.
- The code agreement can either be assessed by independently coding the challenging paper or assessing the original reviewer's coding.
- Both/all (if >2) reviewers of each paper will meet after the audits are completed to reach a consensus on how to code these challenging papers. Agreement between all reviewers of each paper will be assessed and if no clear consensus can be reached, all team members will discuss until an agreement is reached.
- If paper is too challenging for anyone - removed from analysis as not confident of results.

A summary meeting was also held for each team to check all members had audited consistently and still agreed on the interpretation of the audit framework. If any differences in interpretation were highlighted, the project leader decided on the correct interpretation and audits were rechecked following the discussion, updated, and resubmitted to the project leader.

All final results were sent to the project leader following these field specific summary meetings.

Detailed reviewer instructions

Follow the exclusion criteria and challenging papers protocol (these are detailed above).

Table of which model to choose per field. This is slightly different for each field with the aim to typically choose the ‘main’ model of a paper in a consistent and repeatable manner based on standard format of papers in each field.

A model is defined here as something that draws inference beyond the sample data using quantitative methods (this will include theoretical models but exclude any descriptive statistics) and is explicit in the methods or models part of the paper.

Table: Details of which model to be assessed in multi-model papers in each field

**Field**	**Which model?**	**Comments**
Ecology	Last model in methodology	
Neuroscience	First model in methodology	
Politics	First model in methodology	
Climate Science	First model mentioned in abstract	Must also run analyses in the focal paper i.e. not only use existing model output. If the first model does not meet this criteria - keep going through abstract in order until one does.
Oceanography	Last model in methodology	
Evolution	Last model in methodology	
Geography	Last model in methodology	
Health Science	Start at 1, if this criteria is not available, move to 2 etc.<br>1. “Primary analysis”<br>2. Analysis of the “primary outcome”<br>3. First explicit model after descriptive analysis	

Detailed instructions for the data capture table:

Table: Detailed reviewer instructions in the data capture table

#	Question	Explanation of the question	Answer (expected responses)	Details (expected responses)	Location of uncertainty reporting (expected responses)	Presentation of uncertainty (expected responses)	Model type
1	Is uncertainty in the response included in the focal model?	This question asks you to focus on the response variable of the model you are assessing and report if any uncertainty in that response is included and then reported in some way (i.e. so you can actually identify it)	**Yes/No/NA**<br><br>**Yes** if there is uncertainty reported and it is accounted for in the focal model e.g. measurement error is quantified, the variable is corrected and then used in the model. A Cox model with censoring would be a Yes. If a random effect model is used where measurement error can be distinguished from other patterns, this is a Yes answer as well. (Should be explained in the paper that the random effect structure is used to look at uncertainty).<br><br>**No** if there is no uncertainty reported or if it is not explicitly accounted for in the model. This should be the answer even if the uncertainty CAN'T be quantified. That will be covered in the details column. A linear regression with normal noise and No extra steps will be a No answer here.<br><br>**NA** should be used when the component is not present in the model.<br><br>If there are multiple responses and some have uncertainty reported use **Yes** but write **incomplete** in comments.	If **ANSWER = Yes** this should cover the type of uncertainty that has been reported e.g. Standard error (**SE**), Bootstrap interval (**BI**), Credible interval or confidence interval (**CI**), measurement error (**ME**), observation error (**OE**), a state model (**State**).<br><br>Full list of uncertainty types below.<br><br>If **ANSWER = No** this can explain if the uncertainty is Missing (**MISS**), Impossible (**IM**), or no uncertainty in the variable i.e. uncertainty is not relevant for this variable (**NU**), **Not propagated** (if mentioned elsewhere but not included in model)<br><br>Can also include: **NA, I don't know, can't tell**.<br><br>IF ANSWER = NO this can explain if the uncertainty is Missing (**MISS**), Impossible (**IM**), or no uncertainty in the variable i.e. uncertainty is not relevant for this variable (**NU**), I expect NU to be rare, **Not propagated** (if mentioned elsewhere but not included in model)	**Main** = in the main article, **SI** = in supporting information or appendix, **Data** = with a data publication, **Citation** = in another paper that is cited (for citation the focal paper must indicate the uncertainty is reported at the citation and also use it in the model.<br><br>Only give one answer: If multiple apply default to the most accessible (i.e. in order presented here)	Here give details of how it was reported e.g. **visual** (in a figure), **text** (in the main text as words - this can just be authors saying "we did X to quantify uncertainty in Y" but never giving a number), **numeric** (as a number either in a table or text), other.<br><br>Combinations can be used e.g. visualnumeric or visualtext. Please stay in order of **visual, text, numeric**<br><br>Report **all** presentation techniques used.<br><br>Make sure text is selected if the authors write something like "we account for uncertainty in X with Y" just saying we ran a model is not enough for text.	Only answer in this first row for the whole model:<br><br>qualitative, dynamical, mechanistic, statistical, theoretical

2	Is the uncertainty in the explanatory variables included in the focal model?	This question asks you to focus on the explanatory variables of the model you are assessing and report if any uncertainty is included and reported in some way	**Yes/No/NA**<br><br>**Yes** if there is uncertainty reported and it is accounted for in the focal model e.g. measurement error is quantified, the variable is corrected and then used in the model.<br><br>**No** if there is no uncertainty reported and if it is not explicitly accounted for in the model. This should be the answer even if the uncertainty CAN'T be quantified. That will be covered in the details column.<br><br>**NA** should be used when the component is not present in the model.<br><br>If there are multiple explanatory variables and some have uncertainty reported use **Yes** but write **incomplete** in comments.	If **ANSWER = Yes** this should cover the type of uncertainty that has been reported e.g. Standard error (**SE**), Bootstrap interval (**BI**), Credible interval or confidence interval (**CI**), measurement error (**ME**), observation error (**OE**), a state model (**State**).<br><br>Full list of uncertainty types below.<br><br>If **ANSWER = No** this can explain if the uncertainty is Missing (**MISS**), Impossible (**IM**), or no uncertainty in the variable i.e. uncertainty is not relevant for this variable (**NU**), **Not propagated** (if mentioned elsewhere but not included in model)<br><br>Can also include: **NA, I don't know, can't tell**.	**Main, SI, Data, Citation**<br><br>(same as response instructions)	**visual, text, numeric**<br><br>(same as response instructions)	
3	Is the uncertainty in the parameter estimation included (reported in the paper)?	For the model you are looking at, do they report uncertainty in their estimates or the values of the unknown parameters?	**Yes/No/NA**<br><br>**Yes** if there is uncertainty reported and it is accounted for in the focal model e.g. measurement error is quantified, the variable is corrected and then used in the model.<br><br>**No** if there is no uncertainty reported and if it is not explicitly accounted for in the model. This should be the answer even if the uncertainty CAN'T be quantified. That will be covered in the details column.<br><br>**NA** should be used when the component is not present in the model.<br><br>If there are multiple parameters and some have uncertainty reported use **Yes** but write **incomplete** in comments.	If **ANSWER = Yes** this should cover the type of uncertainty that has been reported e.g. Standard error (**SE**), Bootstrap interval (**BI**), Credible interval or confidence interval (**CI**), range of values used as parameter values (**range**).<br><br>Full list of uncertainty types below.<br><br>If **ANSWER = No** this can explain if the uncertainty is Missing (**MISS**), Impossible (**IM**), or no uncertainty in the variable i.e. uncertainty is not relevant for this variable (**NU**), **Not propagated** (if mentioned elsewhere but not included in model)<br><br>Can also include: **NA, I don't know, can't tell**.			

4	Is the uncertainty in the model structure included (reported in the paper)?	For the model you are looking at, do the authors consider how other model types/structures etc might have performed? Or give any range of answers?<br><br>This question goes beyond the focal model as it can include comparison to other models	**Yes/No/NA**<br><br>**Yes** if there is uncertainty reported and it is accounted for in the focal model e.g. measurement error is quantified, the variable is corrected and then used in the model.<br><br>**No** if there is no uncertainty reported and if it is not explicitly accounted for in the model. This should be the answer even if the uncertainty CAN'T be quantified. That will be covered in the details column.<br><br>**NA** should be used when the component is not present in the model.<br><br>Can be Yes for sensitivity analysis that looks at model structure but not for those looking at sub-groups only e.g. removing smokers.	If **ANSWER = Yes** this should cover the type of uncertainty that has been reported e.g. Model comparison (**MC**), model averaging (**MA**), Ensemble (**Ensemble**)<br><br>Full list of uncertainty types below.<br><br>If **ANSWER = No** this can explain if the uncertainty is Missing (**MISS**), Impossible (**IM**), or no uncertainty in the variable i.e. uncertainty is not relevant for this variable (**NU**), **Not propagated** (if mentioned elsewhere but not included in model)<br><br>Can also include: **NA, I don't know, can't tell**.<br><br>Model selection also does not count unless results compared or combined i.e. **model averaging**			
5	Is there an explicit model that maps an unobserved response to observed data? (response)	If the response being modelled is not the same thing the authors had data for then do they map the data and desired response to each other with a model? An example of this would be survival analysis. The data is often recapture (whether you saw an individual again or not), but the model tries to explain survival. Therefore, you have a model that includes both recapture and survival probability explicitly - this would give a **Yes** here.<br><br>Might expect many **No** answers.	**Yes/No**	Give some detail e.g. the process that is unobserved like: **survival**.	NA	NA	
6	Is uncertainty in this element included?	If they model something unobserved, do they explicitly account the uncertainty from this? And the uncertainty associated. If the question above was Yes, the answer to this will be the same as Qu1.	**Yes/No/NA**<br><br>If the answer to Qu5 is No, the answer here should always be NA	Details of uncertainty type e.g. **state** = full state model with reported uncertainty in the response, **ME** = measurement error, **OE** = observation error, **other, NA, I don't know, I can't tell**.	**Main, SI, Data, Citation**<br><br>(same as response instructions)	**visual, text, numeric**<br><br>(same as response instructions)	
7	Do they predict?	Are any predictions made during the final	**Yes/No**		NA	NA	

		analysis or from the model in the final analysis? Predictions are defined as estimating/calculating values of the response outside of the data used in the model.Temporal element of past or future is not important here. Projections would count **if** generated by the focal model in the focal paper.					
**8**	Is uncertainty in the prediction included?	Only answer if question above = Yes	**Yes/No/NA**<br><br>If the answer to Qu7 is No, the answer here should always be NA	Type of uncertainty e.g. prediction interval (**PI**), **SE**, **CI**, **Range** = range of scenarios,<br><br>If **ANSWER = No** can explain why e.g. **not propagated**<br><br>Also, **NA, I don't know, I can't tell**	**Main, SI, Data, Citation**<br><br>(same as response instructions)	**visual, text, numeric**<br><br>(same as response instructions)	
**9**	Is uncertainty from the f**ocal model** discussed in the discussion?	Is any uncertainty discussed in the discussion? This focuses on the discursive part of a paper, often the 'discussion or conclusion' section. You should look for whether the meaning of the uncertainty is included or any reference to how it impacts conclusions. This is not the same as the raw reporting of uncertainty in a results section.<br><br>**This question concerns any uncertainty that was quantified above, not unquantified uncertainty.**<br><br>Potential search words to help identify uncertainty in the discussion: uncertain, overlap, not clear, clear, range, ±	**Yes =** uncertainty is discussed in the paper beyond just reporting. i.e discuss implications.<br><br>**No** = the uncertainty is not discussed after results presented.<br><br>**NA** = to be used if no uncertainty was quantified.	Which part e.g.<br><br>**Response, explanatory, parameter, model, unobserved, prediction, all, none**.			

Uncertainty type definitions:

This whole audit focuses on quantitative uncertainty.

- **Standard error** (**SE**): a standard error from a statistical model. This can be a standard error of a mean, of a difference, etc.
- **Bootstrap interval (BI)**: an interval of confidence (say 95% but could be other levels) obtained by bootstrapping (resampling data and re-running an analysis).
- **Credible interval or confidence interval (CI)**: an interval indicating plausible values usually of an unknown parameter but can also cover values of a latent response or explanatory variable.
- **Measurement error (ME)**: error or bias coming from the measurement of a variable. Should be quantified using an error in the variables model or other appropriate model.
- **Observation error (OE)**: error from observing a process. Should be quantified using an observation model.
- **A state model (State)**: coupled with an observation model, this part models the underlying process of interest and is linked to the observation model.
- **Multiple scenarios (scenarios)**: running an analysis to cover multiple scenarios to give a range of results indicating uncertainty in either model structure, parameters, or variables.
- **Model averaging (MA):** taking a selection of models and averaging parameter estimates. This represents uncertainty introduced by model structure.
- **Model ensemble (En**): taking a collection of models with different structures and presenting results from all of them simultaneously with uncertainty bands reflective of all models in the ensemble. Represents some uncertainty in model structure.
- **Model comparison (MC):** running models with different structures or parameter values or inputs and comparing the results to give an indication of the uncertainty introduced by the varied component.
- **Prediction interval (PI**): an interval indicating a level of uncertainty in a prediction (usually of a response).
- **Range:** model run using a variety of values for that source e.g. using a sequence of parameter values or selecting parameter values randomly from distributions.
- **Standard deviation (SD):** but only of model outputs or estimated parameters, not of observed data (in this later case it just shows variability).
- **Censoring (Censoring):** for example for survival data where the outcome of certain individuals is not known.
- **Full distribution:** using a full posterior distribution from a Bayesian analysis.
- **Hierarchical partitioning:** partitions variance explained among independent variables through assessing the contributions of all orders of the variables and calculating a partition of the variance. Calculates the contribution of each independent variable separately and in conjunction with other variables.

While confidence intervals or a standard error could be calculated using a combination of a p-value and an F-value, this was not considered reporting of uncertainty for this assessment. Presentation of only a p-value and F-value puts the onus on the reader to turn this into a metric of uncertainty. However, it should be noted that providing these details would allow uncertainty to be included in further work, such as meta-analyses, using that paper's results and therefore is still valuable.

Data cleaning

After submission of final results, all data tables were inspected manually first to:

- Remove any blank lines between papers in the table
- Remove example papers
- Correct any obvious spelling mistakes

- Ensure that Paper and Initials columns were filled in for all 9 questions (sometimes only the first was used)
- Check for any Excel errors e.g. increasing year for the Paper ID (2019,2020,2021)
- See if any checks had been tagged as violated to go back to reviewer for correction
- Expand model type to all rows
- Some model types were written in the wrong column – these were moved to the correct one
- All excluded papers were removed from the datafile after exclusion reason was recorded

After manual inspection the data were then imported to R and further checks initiated:

- Check 1 = when Answer = No, Location is NA
- Check 2 = when Answer to Number 1-4, 6 or 8-9 is Yes that Details are not NA
- Check 3 = when Answer = No, Presentation = NA
- Check 4 = when Location is not NA, presentation is not NA
- Check 5 = if the Answer to 5 or 7 is "No" then Answer to 6 and 8 = "NA"
- Check 6 = if Answer for 1-4 is "No", then Answer for 9 = "NA"

If any of these checks are violated the individual rows are checked manually. If the error is obvious and objective e.g. a clear spelling error, this is corrected by the lead author. If there is any ambiguity in the cause of the error, the row is highlighted for the original reviewer and they are asked to check.

If on reading results, any seemed surprising e.g. no parameter uncertainty for a statistical model despite uncertainty being considered for the response and covariates, these papers were reassessed by the original reviewer in discussion with the project leader, or by the project leader alone to ensure consistency across the whole project.

Other errors and how they are dealt with:

Table: Detail of data errors and solutions

**Error**	**Solution**
Wrote out full word when it should be an acronym	Overwrite to the acronym in R
Spelling error	Overwrite in R
Specific location rather than code of Main, SI etc or synonym e.g. appendix	Corrected to the expected code - all cases were unambiguous e.g. table 1
Inclusion of type of uncertainty that was not appropriate e.g. AIC for model structure	Paper was rechecked by lead author and if no qualifying uncertainty type for the component was identified this was corrected to Answer = No

More than one answer when it should just be one e.g. “Main/SI”	Corrected to “Main” only
Syntax errors e.g. capitalisation or inconsistent use of spaces<br><br>Missing model type	Corrected in R<br><br>If clear from comments what the model was, model type was added otherwise left as NA

Data analysis

Data were summarised as the percentage of papers that met a particular criterion. All data analyses were conducted in R (version 4.2.0 [1]).

References

1. R Core Team (2022). R: A Language and Environment for Statistical Computing.
